# Changes in Profiles of Classes and of Individual Polyphenols in Leaves of *Spiraea chamaedryfolia* and *Spiraea media* along an Altitudinal Gradient

**DOI:** 10.3390/plants12162977

**Published:** 2023-08-17

**Authors:** Irina G. Boyarskikh, Igor A. Artemov, Alexander A. Kuznetsov, Vera A. Kostikova

**Affiliations:** 1 Central Siberian Botanical Garden, Siberian Branch of Russian Academy of Sciences, Novosibirsk 630090, Russia; irina_2302@mail.ru (I.G.B.);; 2 Institute of Soil Science and Agrochemistry, Siberian Branch of Russian Academy of Sciences, Novosibirsk 630090, Russia; 3 Laboratory Herbarium (TK), Tomsk State University, Tomsk 634050, Russia; ys.tsu@mail.ru

**Keywords:** *Spiraea*, Republic of Altai, HPLC, flavonoid, phenolcarboxylic acid, natural population, altitudinal gradient

## Abstract

Plants in high-altitude habitats are exposed to severe environmental stressors, including extreme temperatures and irradiation, which can have wide-ranging effects on changes of secondary-metabolite profiles in higher plants. Altitude-related variation of levels of polyphenols in organs of medicinal and food plant species has not yet been investigated sufficiently. This study was focused on variation in quantitative profiles of classes and of individual biologically active phenolic compounds in leaf extracts of resource species *Spiraea chamaedryfolia* and *Spiraea media* from the family Rosaceae in coenopopulations of the Altai Mountains, along an altitudinal gradient. High-performance liquid chromatography revealed 22 polyphenolic compounds in the extracts of *S. media* leaves, with the main polyphenolic compounds being flavonols. Sixteen compounds were found in *S. chamaedryfolia* leaf extracts, and the major ones were flavonols and a flavanone. Opposite responses to changes in the altitude-associated growth conditions were documented for levels of some individual polyphenolic compounds. With an increase in altitude, concentrations of chlorogenic acid and of flavanone in the extracts of *S. chamaedryfolia* leaves significantly increased, while concentrations of cinnamic acid, astragalin, and kaempferol diminished. A statistically significant positive correlation between the altitude of plant habitats and total levels of polyphenols and phenolcarboxylic acids was detected. In leaf extracts from *S. media*, an altitude increase was significantly positively correlated with astragalin, avicularin, and cinnamic acid levels and negatively correlated with hyperoside concentration.

## 1. Introduction

In high-altitude conditions, plants are exposed to several adverse factors, such as low atmospheric pressure, low air temperature, strong winds, a short growing season, and high-intensity ultraviolet radiation [1]. An altitude change affects a wide range of environmental conditions of plant habitats, such as hydrothermal and mineral composition of soils, which directly or indirectly influence levels of secondary metabolites [2,3,4]. The survival of plants under extreme growth conditions depends on their physiological adaptation to various adverse factors that cause oxidative stress. In plants, the defensive mechanism that counteracts oxidative stress consists of the synthesis of antioxidant-defense components, including biologically active phenolic compounds (PCs) [5,6]. Furthermore, shifts in the profile of PCs along an altitudinal gradient may be species-specific [7]. In some studies on variation in profiles of classes and of individual PCs in leaf extracts from *Lonicera caerulea* L. (Caprifoliaceae Juss.) in populations of the Altai Mountains (Seminsky Ridge), it has been found that various PC classes and individual PCs are characterized by different directions of the response to shifts of growth conditions along the altitudinal gradient [8].

Ethnomedical use of species of the genus *Spiraea* L. (family Rosaceae Juss.) has been documented in North America, Russia, and Asia [9,10,11]. *Spiraea* species are used as effective pain relievers and anti-inflammatory, detoxifying, diuretic, and other remedies. The therapeutic use of *Spiraea* species in folk medicine has been validated by modern scientific experiments [12]. *Spiraea chamaedryfolia* L. is a shrub up to 150 cm tall. In leaves of *S. chamaedryfolia*, researchers have found oxybenzoic acids (gallic and protocatechuic) and hydroxycinnamic acids (chlorogenic, caffeic, and *n*-coumaric) as well as flavonoids (hyperoside and isoquercitrin) [13]. In roots of *S. chamaedryfolia*, there are alkaloids that accumulate in the secondary bark and secondary xylem of the root, while alkaloids are absent in the core [14]. *S. chamaedryfolia* extracts of different polarity have high inhibitory activity against xanthine oxidase (>70%) and moderate antibacterial activity against *Staphylococcus aureus*, *Bacillus subtilis*, *Streptococcus pneumoniae*, and *Moraxella catarrhalis* [15]. Aqueous-ethanol extracts from *S. chamaedryfolia* have moderate antiviral activity toward the A/H5N1 avian influenza virus [16]. *Spiraea media* F. Schmidt is a shrub up to 2 m tall. In leaves of *S. media*, quercetin, kaempferol, isorhamnetin, and their glycosides as well as phenolcarboxylic acids have been detected [17,18]. In leaves and inflorescences of *S. media*, high concentrations of catechins (up to 57 mg/g) and saponins (up to 30 mg/g) have been found [19,20]. *S. media* has antioxidant and antiviral properties and a protistocidal effect against *Paramecium caudatum* [16,21,22,23]. An aqueous-ethanol extract from the leaves of *S. media* exerts an anti-inflammatory effect in vivo [24].

Both *Spiraea* species analyzed in the present work (*S. chamaedryfolia* and *S. media*) belong to the section *Chamaedryon* Ser. *Spiraea* species of this section are characterized by simple corymbose or umbelliform inflorescences at the ends of lateral leafy shoots [25]. The species under study are well distinguished from each other morphologically: *S. chamaedryfolia* has broadly or oblongly ovate leaves with a duplicato-dentate margin almost from the base, whereas *S. media* has elliptical oblong leaves with three denticles at the apex [25,26,27].

The purpose of the current work was a comparative study on populational variation in profiles of biologically active PCs and of their classes in leaf extracts of two *Spiraea* species (*S. media* and *S. chamaedryfolia*) in coenopopulations of the Altai Mountains in a Multa River valley (Altai Republic) along an altitudinal gradient.

## 2. Results and Discussion

We performed a comparative examination of retention times of the peaks of the compounds in chromatograms of the analyzed and standard samples; we also carried out a computational comparison of the absorption spectra obtained by chromatography of the substances with a library that we have. As a result, profiles of classes of PCs and of these individual compounds were determined in the aqueous-ethanol extracts from the leaves of the plant species being studied: *S. chamaedryfolia* and *S. media* (Table 1 and Table 2).

**Figure 1:**
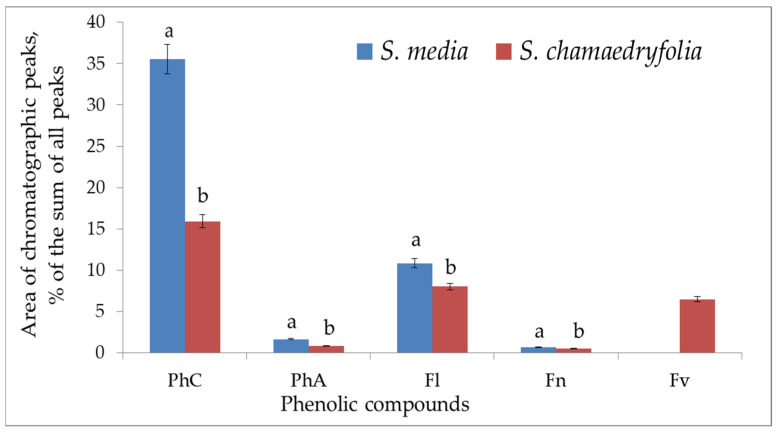
Mean concentrations of classes of PCs in *S. media* and *S. chamaedryfolia* leaf extracts in the coenopopulations growing in the Multa River valley. Vertical axis: area of chromatographic peaks (percentage of the sum of all peaks). Horizontal axis: PCs. PhC: The sum of PCs; PhA: the sum of phenolic acids; Fl: the sum of flavonols; Fn: the sum of flavones; Fv: the sum of flavanones. Different letters indicate significant differences in parameters (*p* ˂ 0.01) according to Student’s *t*-test.

**Figure 2:**
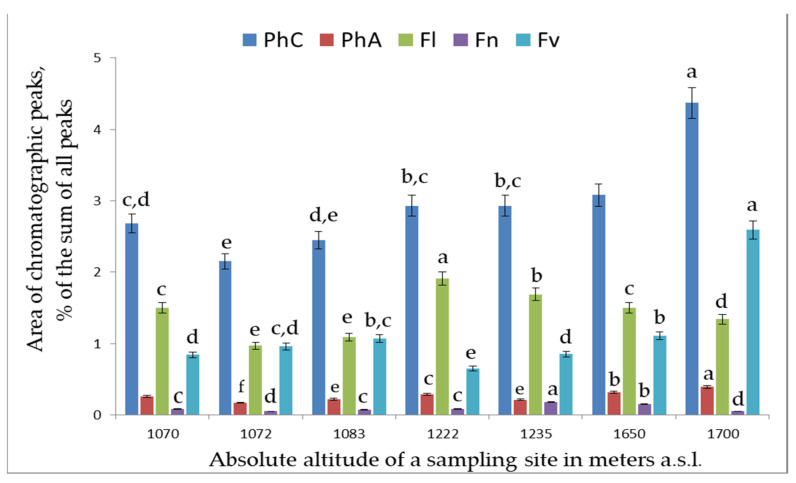
The dependence of concentrations of classes of PCs in extracts from *S. chamaedryfolia* leaves on altitude. Vertical axis: area of chromatographic peaks (percentage of the sum of all peaks). Horizontal axis: absolute altitude of a sampling site in meters a.s.l.; PhC: the sum of PCs; PhA: the sum of phenolic acids; Fl: the sum of flavonols; Fn: the sum of flavones; Fv: the sum of flavanones. Different letters indicate significant differences in parameters (*p* ˂ 0.05) according to Tukey’s HSD test.

**Figure 3:**
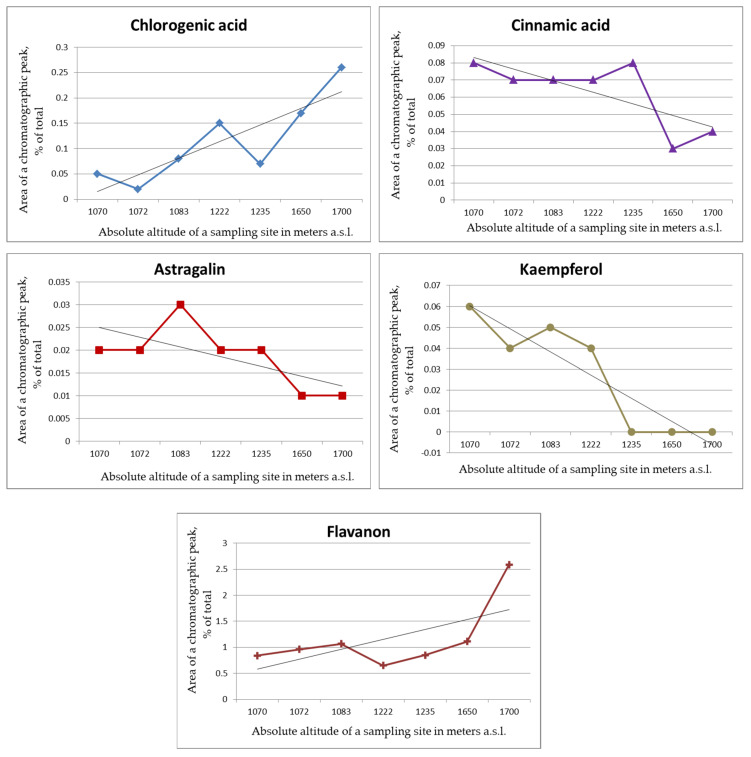
The dependence of concentrations of individual PCs in leaf extracts of *S. chamaedryfolia* on altitude. Horizontal axis: absolute altitude of a sampling site in meters a.s.l.; vertical axis: area of a chromatographic peak (percentage of total).

**Figure 4:**
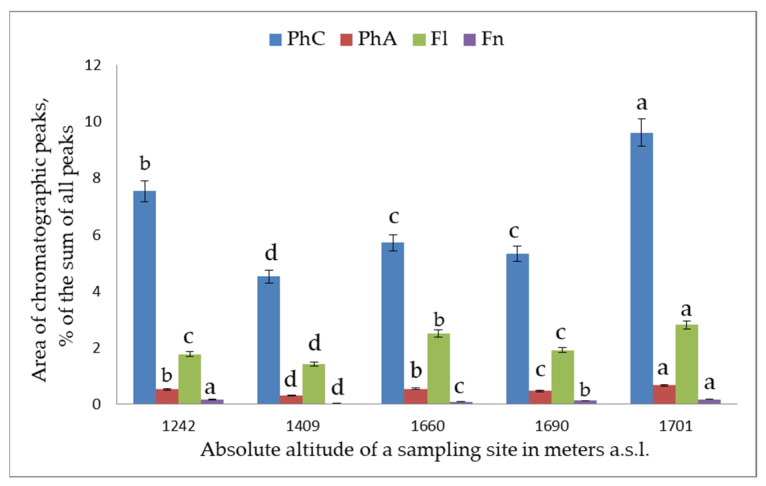
The dependence of concentrations of classes of PCs in extracts from *S. media* leaves on altitude. Horizontal axis: absolute altitude of a sampling site in meters a.s.l.; vertical axis: area of chromatographic peaks (percentage of the sum of all peaks). PhC: the sum of PCs; PhA: the sum of phenolic acids; Fl: the sum of flavonols; Fn: the sum of flavones. Different letters indicate significant differences in parameters (*p* ˂ 0.05) according to Tukey’s HSD test.

**Figure 5:**
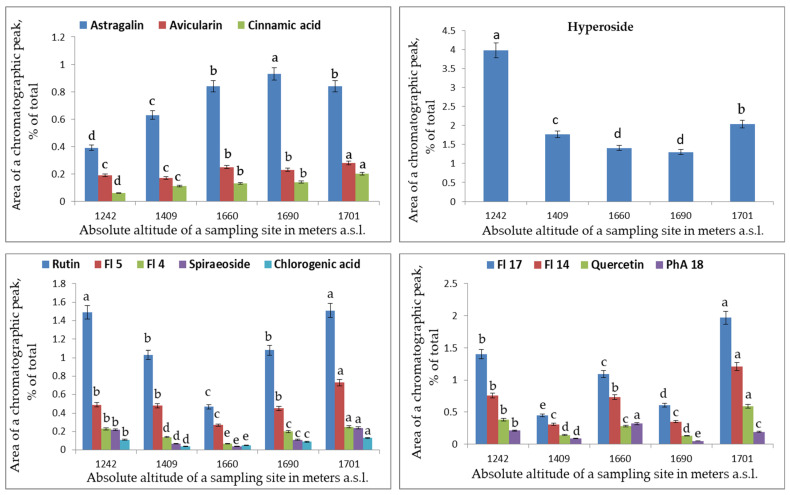
The dependence of levels of individual PCs in leaf extracts of *S. media* on altitude. Horizontal axis: absolute altitude of a sampling site in meters a.s.l.; vertical axis: area of a chromatographic peak (percentage of total). Different letters indicate significant differences in parameters (*p* ˂ 0.05) according to Tukey’s HSD test.

The comparative analysis of the levels of PC classes in the plant leaves showed that the total polyphenol content in *S. media* varied within 24.6–52.4 mg/g, with major PCs being flavonols. *S. chamaedryfolia* is distinguished by a significantly lower concentration of polyphenols (11.7–23.6 mg/g), the main of which are flavonols and flavanones. The analyzed species manifested high variation both of the total content of PCs and of their individual classes and representatives, depending on the sampling site (Figure 1, Figure 2, Figure 3, Figure 4 and Figure 5).

The leaf extracts of *S. chamaedryfolia* were found to contain 16 biologically active PCs (Table 1). Major PCs of the extracts were an unidentified flavanone (retention time [t_R_] = 11.5) and flavonols: rutin, spiraeoside, and an unidentified flavonol (t_R_ = 7.3). In all the studied coenopopulations, chlorogenic and cinnamic acids, an unidentified phenolcarboxylic acid (t_R_ = 44.0), flavones (t_R_ = 4.4 and 23.8), isoquercitrin, and astragalin were detected in minor amounts. In this species, other PCs were found in some coenopopulations only in small amounts. The largest set of polyphenols was detected in leaf extracts of the *S. chamaedryfolia* collected in CP1 and CP2, respectively, at 1070 and 1222 m above sea level (a.s.l.). The highest total content of polyphenols (23.8 mg/g) was noted in CP7 (1700 m a.s.l.), whereas the lowest content (11.7 mg/g) was registered at the lowest location (CP1 at 1072 m a.s.l.).

The levels of individual polyphenols and of their classes in the leaf extracts of *S. chamaedryfolia* varied significantly depending on the location of a coenopopulation (Figure 2 and Figure 3). In terms of these concentrations, we noticed some patterns. Along the gradient of increasing altitude, there was a significant (*p* < 0.01) increase in the level of chlorogenic acid (r = 0.89), and at *p* < 0.05, an increase of flavanone concentration (r = 0.71) and a decrease in concentrations of astragalin (r = −0.87, *p* < 0.01), cinnamic acid (r = −0.91, *p* < 0.01), and kaempferol (r = −0.82, *p* < 0.05). A direct statistically significant (*p* < 0.05) correlation was found between the altitude where the plants grew and the total contents of PCs and of phenolcarboxylic acids in the extracts of *S. chamaedryfolia* leaves (r = 0.84 and 0.85, respectively).

In *S. media* leaf extracts, 22 PCs were found (Table 2). The major ones were flavonols: rutin; hyperoside; quercetin; spiraeoside; astragalin; avicularin; kaempferol; unidentified flavonols 4, 5, 14, and 17 (compound ID numbers in Table 2); and an unidentified flavanone (t_R_ = 11.5). Unidentified flavonols 15, 20, and 21 were present only in minor amounts, whereas in some coenopopulations, the same was true for n-coumaric acid and dihydroquercetin. The largest set of PCs was found in the leaves of *S. media* plants collected in CP2 and CP7, which are the lowest location (for *S. media*) (1242 m a.s.l.) and the highest location (1701 m a.s.l.), respectively. The highest total contents of PCs were also observed in the same habitats (Figure 4): 41.1 and 52.4 mg/g, respectively. The lowest total contents of PCs were observed in coenopopulations located at 1409, 1660, and 1690 m a.s.l. (24.6, 30.9, and 28.8 mg/g, respectively).

With the increasing altitude, the concentration of astragalin (r = 0.98) increased significantly (*p* < 0.001), and at *p* < 0.05, the same was true for avicularin (r = 0.83) and cinnamic acid (r = 0.87). Meanwhile, the hyperoside content diminished significantly (*p* < 0.05; r = 0.82). Various compounds could be grouped according to the nature of their accumulation in the tested coenopopulations (Figure 5). The levels of astragalin, avicularin, and cinnamic acid went up with altitude, while the level of hyperoside decreased.

Patterns of accumulation of rutin, spiraeoside, chlorogenic acid, flavonol 4, and flavonol 5 resembled one another. In the plant leaf extracts, the lowest concentrations of these PCs were detected in CP5 (1660 m a.s.l.; Figure 5). With the increasing distance from this location up and down the altitudinal gradient, the concentration of this set of components increased. Quercetin, phenolic acid 18, and flavonols 14 and 17 also showed patterns similar to one another, but in CP5, there was an increase in their concentrations as compared to neighboring coenopopulations (Figure 5). A possible reason is special micro conditions at this location.

Our results about the profiles of classes of PCs and of individual PCs in leaf extracts from *S. media* and *S. chamaedryfolia* along the altitudinal gradient in the Multa River valley (Altai Mountains) revealed significant variation in concentrations of PC classes and individual PCs. In *S. media* leaves, the main PCs were found to be affiliated with the class of flavonols, whereas in *S. chamaedryfolia* leaves, in addition to flavonols, a high level of flavanone was registered. Different directions of the response of individual PCs to changes in growth conditions (associated with altitude) were documented in our work. For instance, an increase in altitude had a significant positive correlation with the concentration of chlorogenic acid and flavanone in the extracts of *S. chamaedryfolia* leaves and a negative correlation with concentrations of cinnamic acid, astragalin, and kaempferol there. A direct statistically significant correlation was detected between the altitude where the plants grew and the total contents of polyphenols and of phenolcarboxylic acids in this species. In the extracts of *S. media* leaves, as altitude increased, levels of astragalin, avicularin, and cinnamic acid increased too, while the concentration of hyperoside declined. Regarding the variation of concentrations of the other identified compounds in the extracts, no significant dependence on altitude was found (in both species).

The opposite directions of some responses of PC classes and of individual PCs to the changes in growth conditions (associated with altitude) can apparently be explained by the different functions performed by individual polyphenols in the physiological processes of the plants. Many researchers have reported either a positive correlation between altitude and levels of PCs [28,29] or different directions of this correlation for different PC classes and for individual PCs [30,31,32,33]. Previously, we have also noticed different directions of the response of classes of PCs to shifts of growth conditions along an altitudinal gradient in populations of *Lonicera caerulea* in the Altai Mountains [8,34].

The patterns of changes in concentrations of PC classes and of individual PCs along the altitudinal gradient turned out to be different between the two *Spiraea* species analyzed in the current work. These differences between the closely related species are determined by the aforementioned features of the profiles of individual PCs and of their classes and by species-specific optima of growth conditions. It is likely that the different ecological and phytocoenotic affiliations of these species differently affect the synthesis of classes and of individual secondary metabolites in these plants. *S. chamaedryfolia* grows in the forest belt and subalpine vegetation belt in the mountains of Europe, Southern Siberia, Northern China, and Mongolia, where it occurs at up to 2000 m a.s.l. [26,27], in contrast to *S. media*, which has a wider geographic range, including plains. The latter species occurs throughout Russia and in Europe, Central Asia, China, Korea, Japan, and Mongolia [26,27]. In the Altai Mountains, the *Spiraea* species under study differ in growth conditions. *S. chamaedryfolia* is confined more to mountain forest communities, while *S. media* is confined more to shrubby meadow communities. It is likely the differences in the patterns of synthesis of polyphenols between the analyzed *Spiraea* species are also related to the confinement of these species to dissimilar ecological and phytocoenotic conditions.

## 3. Materials and Methods

### 3.1. Plant Material

**Figure 6:**
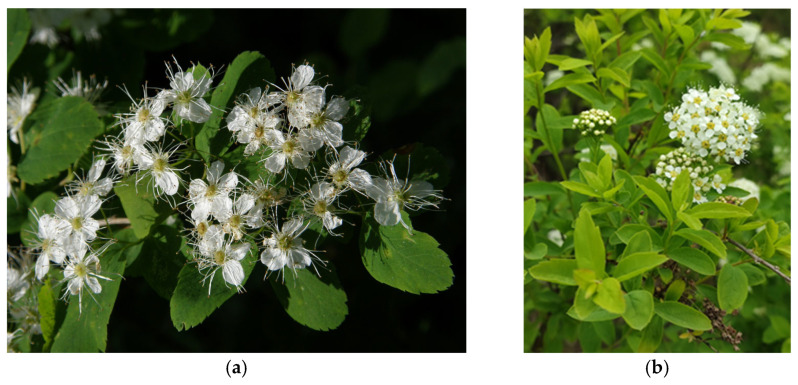
The *Spiraea* species under study: (**a**) *Spiraea chamaedryfolia*; (**b**) *Spiraea media*.

The study was conducted in 2019 in the Republic of Altai, in a valley of the Multa River (Ust-Koksinsky District) within a geobotanical sub-province called the Central Altai. Sample plots were selected within an altitude profile of 1070–1850 m a.s.l.: coenopopulation 1 (CP1): at 1070–1083 m a.s.l. (50°07′ N, 85°57′ E) in a spruce–larch graminaceous forest of various grasses; CP2: at 1222–1242 m a.s.l. (50°05′ N, 85°54′ E) in a birch–spruce–cedar–larch graminaceous forest of various grasses; CP3: at 1409 m a.s.l. (50°04′ N, 85°53′ E) in a birch–spruce–cedar–larch forest; CP4: at 1650–1651 m a.s.l. (50°01′ N, 85°50′ E) in a larch–spruce–cedar graminaceous forest of various grasses; CP5: at 1660–1665 m a.s.l. (49°58′ N, 85°50′ E) in a spruce–cedar–larch graminaceous forest of various grasses; CP6: at 1690 m a.s.l. (49°57′ N, 85°51′ E) in a larch–spruce–cedar graminaceous forest of various grasses; and CP7: at 1700–1701 m a.s.l. (49°56′ N, 85°51′ E) in a larch–spruce–cedar graminaceous forest of various grasses. In the delineated coenopopulations, tissue samples (leaves) of medicinal plants *S. chamaedryfolia* (Figure 6a) and *S. media* (Figure 6b) were collected for the quantification of PCs. The leaves of these *Spiraea* species were collected only from specimens having typical morphological features.

The sampling was carried out simultaneously for the two investigated species at the end of the flowering stage. The plant material was collected for 10 days to ensure that the sampling was performed in the same phase of seasonal plant development. The plant leaves were air-dried completely under natural conditions.

The study sites are located in Katunsky Nature Reserve and its border area; this choice ensured the absence of technogenic pollution and of its possible influence on the variation of a secondary metabolism.

### 3.2. Preparation of the Extract

To assay PCs, aqueous-ethanol (70% ethyl alcohol) extracts from *S. media* and *S. chamaedryfolia* leaves were prepared by means of a water bath. Approximately 1.0 g of the raw material (precise weight was recorded) that passed through a sieve with a pore diameter of 2–3 mm was placed into a 100 mL round-bottom flask with a ground-in stopper. The raw material was covered with 30 mL of 70% ethanol, and the flask was attached to a reflux condenser and placed in a boiling water bath for 30 min incubation. The flask was shaken from time to time to wash particles of the raw material off the walls. After that, the flask with the extract was cooled, and the first portion of the extract was passed through a paper filter into a conical flask (volume 100 mL) with a ground-in stopper. Next, the raw material from the filter was put in a round-bottom flask and again covered with 30 mL of 70% ethanol and extracted for 30 min in the boiling water bath. The second portion of the extract was cooled and filtered into the first portion of the extract in the 100 mL flask. The procedure was repeated one more time. The three portions of the extract were mixed, and the volume of the resulting combined extract was measured.

### 3.3. Determination of the Set and Levels of Individual PCs by High-Performance Liquid Chromatography (HPLC)

One milliliter of each aqueous-ethanol extract was diluted with double-distilled water to 5 mL and passed through a Diapak C16 concentrating cartridge (BioChemMac Corporation, Moscow, Russia). Substances were eluted from the cartridge by means of 3 mL of 70% ethanol and then using 2 mL of 96% ethanol. The combined eluate was passed through a membrane filter with 0.45 μm pore size.

PCs in the eluate were studied by means of an analytical HPLC system consisting of an Agilent 1200 liquid chromatograph (USA) equipped with a diode array detector, an autosampler, and a ChemStation system for the collecting and processing of chromatographic data by a method of T.A. van Beek [35] with modifications, on a Zorbax SB-C18 column (4.6 × 150 mm, 5 μm). The chromatographic analysis was first carried out as isocratic elution in a methanol–0.1% orthophosphoric acid (31:69) system for 27 min, and then in a gradient elution mode: in the mobile phase, the proportion of methanol in the mixture with orthophosphoric acid (0.1% in water) was changed from 33% to 46% for 11 min, then from 46% to 56% during the next 12 min, and finally from 56% to 100% for 4 min. The eluent flow rate was 1 mL/min, column temperature was 26 °C, and injection volume was 10 µL. Detection was carried out at wavelengths (λ) 254, 270, 290, 340, 360, and 370 nm.

The quantification of PCs was conducted as reported previously [36]. The quantitative analysis of individual PCs in the plant samples was performed by the external standard method at λ = 360 nm. Standard samples were prepared from cinnamic acid (Serva Heidelberg, Germany), chlorogenic and *p*-coumaric acids, quercetin, and kaempferol (Sigma-Aldrich Taufkirchen, Germany), isoquercitrin, rutin, avicularin, spiraeoside, and astragalin (Fluka, Chemie AG, Buchs, Switzerland), and dihydroquercetin (Austrowaren, Austria). By means of spectral characteristics, unidentified compounds were assigned to a class of PCs according to ultraviolet spectral characteristics from the literature [37,38,39]. The calculation of phenolic acids’ concentrations was carried out on the basis of chlorogenic acid, and the calculation of flavonoids’ concentrations on the basis of rutin.

The relative standard deviation (σ_r,rel_, a metric of repeatability) of the quantification of PCs was 0.011; the relative standard deviation in terms of retention time in the HPLC was 0.0018.

### 3.4. Statistical Analysis

Statistical processing of the experimental data was carried out using the STATISTICA 6.1 software package. Correlation analysis was performed by the Pearson method; critical significance levels are given in ref. [40]. Student’s *t*-test was performed to determine the statistical significance of a difference in substance levels between specimens of *Spiraea* species. Multiple comparisons were performed by one-way ANOVA followed by Tukey’s honestly significant difference (HSD) test to evaluate the significance of differences among the means [40]. The significance level was set to *p* < 0.01 and 0.05, and the data are presented as mean ± standard deviation.

## 4. Conclusions

A comparative study on the variation in quantitative profiles of classes and of individual biologically active PCs in leaf extracts from resource species *S. chamaedryfolia* and *S. media* in coenopopulations of the Altai Mountains in the Multa River valley showed a significant variation of secondary-metabolites’ concentrations depending on the sampling site along an altitudinal gradient. In *S. chamaedryfolia* leaf extracts, the total content of polyphenols and phenolcarboxylic acids significantly increased with increasing altitude, mainly owing to the significant upregulation of chlorogenic acid and flavanone. Levels of cinnamic acid, astragalin, and kaempferol significantly diminished with altitude. For *S. media*, an increase in altitude correlated significantly positively with concentrations of astragalin, avicularin, and cinnamic acid in the leaf extracts and correlated negatively with the hyperoside level. The species-specific patterns of changes in PCs’ levels along the altitudinal gradient are probably determined by the confinement of these species to different ecological and phytocoenotic conditions.

## Figures and Tables

**Table 1:** Variation of individual PCs’ levels (mean ± SD, mg/g of air-dry mass of raw material) in the extracts from *S. chamaedryfolia* leaves depending on altitude.

Peak ID Number	Compound	Retention Time, min	Spectral Characteristics, λ_max_, nm	Altitude, m a.s.l.						
				1070	1072	1083	1222	1235	1650	1700
1	Chlorogenic acid	3.2	244, 330	0.17 ± 0.01 ^d^	0.07 ± 0 ^e^	0.26 ± 0.01 ^c^	0.5 ± 0.02 ^b^	0.22 ± 0.01 ^c^	0.54 ± 0.02 ^b^	0.86 ± 0.03 ^a^
2	Flavone	4.4	270, 320	0.09 ± 0 ^a^	0.08 ± 0 ^b^	0.09 ± 0 ^a^	0.08 ± 0 ^b^	0.08 ± 0 ^b^	0.08 ± 0 ^b^	0.1 ± 0 ^c^
3	Flavonol	7.3	255, 355	3.45 ± 0.13 ^d^	1.08 ± 0.04 ^f^	2.2 ± 0.08 ^e^	7.5 ± 0.28 ^a^	6.43 ± 0.24 ^b^	5.23 ± 0.19 ^c^	3.69 ± 0.14 ^d^
4	Flavanone	11.5	285, 335	4.74 ± 0.18 ^c^	5.37 ± 0.20 ^c^	6.02 ± 0.22 ^b,c^	3.67 ± 0.14 ^d^	4.76 ± 0.18 ^c^	6.2 ± 0.23 ^b^	14.55 ± 0.54 ^a^
5	Flavonol	15.2	250, 355	0.21 ± 0.01 ^b^	0.28 ± 0.01 ^a^	0.09 ± 0 ^c^	0.08 ± 0 ^d^	0.08 ± 0 ^d^	0	0
6	Isoquercitrin	19.3	255, 355	0.42 ± 0.02 ^d^	0.58 ± 0.02 ^c^	0.41 ± 0.02 ^d,e^	0.35 ± 0.01 ^e^	0.85 ± 0.03 ^a^	0.57 ± 0.02 ^c^	0.72 ± 0.03 ^b^
7	Rutin	20	256, 358	2.18 ± 0.08 ^b^	2.41 ± 0.01 ^a^	2.03 ± 0.08 ^b,c^	0.65 ± 0.02 ^f^	0.92 ± 0.03 ^e^	1.28 ± 0.05 ^d^	1.97 ± 0.07 ^c^
8	Flavone	23.8	250, 340	0.34 ± 0.01 ^c^	0.24 ± 0.01 ^d^	0.28 ± 0.01 ^d^	0.39 ± 0.01 ^c^	0.96 ± 0.04 ^a^	0.78 ± 0.03 ^b^	0.15 ± 0.01 ^e^
9	Spiraeoside	26.8	255, 365	1.05 ± 0.04 ^b^	0.68 ± 0.03 ^d^	0.86 ± 0.03 ^c^	0.57 ± 0.02 ^e^	1.02 ± 0.04 ^b^	1.3 ± 0.05 ^a^	1.07 ± 0.04 ^b^
10	Astragalin	32.5	265, 350	0.14 ± 0.01 ^a^	0.12 ± 0 ^b^	0.14 ± 0.01 ^a^	0.12 ± 0 ^b^	0.13 ± 0 ^a,b^	0.04 ± 0 ^d^	0.05 ± 0 ^c^
11	Cinnamic acid	35.9	216, 270	0.25 ± 0.01 ^a,b^	0.22 ± 0.01 ^c^	0.24 ± 0.01 ^b,c^	0.23 ± 0.01 ^b,c^	0.27 ± 0.01 ^a^	0.1 ± 0 ^d^	0.11 ± 0.00 ^d^
12	Flavonol	38.5	265, 355	0.12 ± 0 ^b^	0	0	0.3 ± 0.01 ^a^	0	0	0
13	Quercetin	40.6	255, 372	0.15 ± 0.01 ^b^	0.1 ± 0 ^e^	0.12 ± 0 ^c^	0.32 ± 0.01 ^a^	0.11 ± 0 ^d^	0	0
14	Flavonol	41.9	250, 360	0.36 ± 0.01 ^b^	0	0	0.61 ± 0.02 ^a^	0	0	0
15	Phenolcarboxylic acid	44	235, 315	0.42 ± 0.02 ^a^	0.25 ± 0.01 ^d^	0.23 ± 0.01 ^d,e^	0.23 ± 0.01 ^d,e^	0.2 ± 0.01 ^e^	0.38 ± 0.01 ^b^	0.3 ± 0.01 ^c^
16	Kaempferol	46.9	266, 370	0.32 ± 0.01 ^a^	0.23 ± 0.01 ^c^	0.29 ± 0.01 ^b^	0.22 ± 0.01 ^c^	0	0	0

Note. Means followed by different letters in the same column are significantly different (*p* ˂ 0.05) according to Tukey’s HSD test.

**Table 2:** Variation of levels of individual PCs (mean ± SD; mg/g of air-dry mass of raw material) in extracts from *S. media* leaves depending on altitude.

Peak ID Number	Compound	Retention Time, min	Spectral Characteristics, λ_max_, nm	Altitude, m a.s.l.				
				1242	1409	1660	1690	1701
1	Chlorogenic acid	3.2	244, 330	0.36 ± 0.01 ^b^	0.14 ± 0.01 ^e^	0.18 ± 0.01 ^d^	0.31 ± 0.01 ^c^	0.42 ± 0.02 ^a^
2	*p*-Coumaric acid	7.9	226, 320	0.3 ± 0.01 ^b^	0	0	0.36 ± 0.01 ^a^	0.04 ± 0 ^c^
3	Dihydroquercetin	8.5	290	0.32 ± 0.01 ^c^	0.5 ± 0.02 ^a^	0.39 ± 0.01 ^b^	0	0.15 ± 0.01 ^d^
4	Flavonol	10.9	255, 360	1.28 ± 0.05 ^b^	0.79 ± 0.03 ^d^	0.42 ± 0.02 ^e^	1.11 ± 0.04 ^c^	1.41 ± 0.05 ^a^
5	Flavonol	15.2	250, 355	2.74 ± 0.10 ^b^	2.69 ± 0.10 ^b^	1.5 ± 0.06 ^c^	2.53 ± 0.09 ^b^	4.07 ± 0.15 ^a^
6	Hyperoside	18	255, 355	3.98 ± 0.15 ^a^	1.77 ± 0.07 ^c^	1.41 ± 0.05 ^d^	1.3 ± 0.05 ^d^	2.04 ± 0.08 ^b^
7	Rutin	20	256, 358	8.36 ± 0.31 ^a^	5.79 ± 0.21 ^b^	2.63 ± 0.10 ^c^	6.08 ± 0.22 ^b^	8.46 ± 0.31 ^a^
8	–	23	–	0.87 ± 0.03 ^b^	0.84 ± 0.03 ^b^	0.58 ± 0.02 ^c^	0.89 ± 0.03 ^b^	1.74 ± 0.06 ^a^
9	Flavone	23.8	250, 340	0.96 ± 0.04 ^a^	0.25 ± 0.01 ^d^	0.48 ± 0.02 ^c^	0.75 ± 0.03 ^b^	1.03 ± 0.04 ^a^
10	Spiraeoside	26.8	255, 365	1.26 ± 0.05 ^a^	0.37 ± 0.01 ^c^	0.23 ± 0.01 ^d^	0.59 ± 0.02 ^b^	1.32 ± 0.05 ^a^
11	Avicularin	28.4	260, 360	1.08 ± 0.04 ^c^	0.95 ± 0.04 ^c^	1.4 ± 0.05 ^b^	1.3 ± 0.05 ^b^	1.56 ± 0.06 ^a^
12	Astragalin	32.5	265, 350	2.17 ± 0.08 ^d^	3.54 ± 0.13 ^c^	4.72 ± 0.17 ^b^	5.24 ± 0.19 ^a^	4.74 ± 0.18 ^b^
13	Cinnamic acid	35.9	216, 270	0.21 ± 0.01 ^d^	0.35 ± 0.01 ^c^	0.42 ± 0.02 ^b^	0.44 ± 0.02 ^b^	0.65 ± 0.02 ^a^
14	Flavonol	38.5	265, 355	4.26 ± 0.16 ^b^	1.76 ± 0.07 ^c^	4.12 ± 0.15 ^b^	1.95 ± 0.07 ^c^	6.77 ± 0.25 ^a^
15	Flavonol	39.7	260, 360	0.62 ± 0.02 ^a^	0.2 ± 0.01 ^d^	0.51 ± 0.02 ^b^	0.17 ± 0.01 ^d^	0.25 ± 0.01 ^c^
16	Quercetin	40.6	255, 372	2.15 ± 0.08 ^b^	0.81 ± 0.03 ^d^	1.58 ± 0.06 ^c^	0.75 ± 0.03 ^d^	3.28 ± 0.12 ^a^
17	Flavonol	41.9	250, 360	7.86 ± 0.29 ^b^	2.5 ± 0.09 ^e^	6.09 ± 0.23 ^c^	3.41 ± 0.13 ^d^	11.05 ± 0.41 ^a^
18	Phenolcarboxylic acid	42.9	255, 265, 315	0.69 ± 0.03 ^a^	0.29 ± 0.01 ^d^	0.47 ± 0.02 ^c^	0.15 ± 0.01 ^e^	0.63 ± 0.02 ^b^
19	Phenolcarboxylic acid	44	235, 315	0.2 ± 0.01 ^d^	0.22 ± 0.01 ^d^	0.75 ± 0.03 ^a^	0.27 ± 0.01 ^c^	0.45 ± 0.02 ^b^
20	Flavonol	45.7	270, 350	0.79 ± 0.03 ^b^	0.2 ± 0.01 ^d^	0.56 ± 0,02 ^c^	0.08 ± 0 ^e^	0.96 ± 0.04 ^a^
21	Flavonol	46	270, 355	0.29 ± 0.01 ^b^	0.06 ± 0 ^d^	0.4 ± 0,01 ^a^	0.08 ± 0 ^c^	0.3 ± 0.01 ^b^
22	Kaempferol	46.9	266, 370	0.33 ± 0.01 ^d^	0.56 ± 0.02 ^c^	2.05 ± 0.08 ^a^	1 ± 0.04 ^b^	1.04 ± 0.04 ^b^

Note. “–”: the compound could not be identified. Means followed by different letters in the same column are significantly different (*p* ˂ 0.05) according to Tukey’s HSD test.

## Data Availability

Raw data of this study are available upon request from the corresponding author.
